# Temporal-Comparative Feedback Facilitates Golf Putting

**DOI:** 10.3389/fpsyg.2018.02691

**Published:** 2019-01-04

**Authors:** Suzete Chiviacowsky, Natália Maass Harter, Gisele Severo Gonçalves, Priscila Lopes Cardozo

**Affiliations:** Department of Physical Education, Universidade Federal de Pelotas, Pelotas, Brazil

**Keywords:** self-evaluation, perceived competence, feedback, motivation, golf

## Abstract

The present experiment investigated the influence of temporal-comparative feedback in young adults learning a sport motor skill. A positive temporal-comparative feedback group and a control group practiced putting golf balls to a target from a distance of 150 cm and received augmented feedback (deviation in cm) in addition to their intrinsic visual feedback. The temporal-comparative feedback group was given additional feedback after each block of 10 trials, suggesting that their average performance was better than it was in the previous block. One day after the practice phase a retention test was performed, to observe learning effects. The results showed that positive temporal-comparative feedback enhances the learning of a putting golf task. Greater putting accuracy was observed on the retention test for the temporal comparison group relative to the control group. Questionnaire results also indicated that participants in this group reported higher perceived competence at the end of practice relative to control participants. The findings provide further indication that temporal comparison affects the learning of motor skills and highlight the important motivational role of feedback in motor learning.

## Introduction

In recent years, an increasing number of studies have investigated the effects of three important motivational variables (Deci and Ryan, 2000) on motor learning. The provision of competence (Chiviacowsky and Wulf, 2007; Clark and Ste-Marie, 2007; Lewthwaite and Wulf, 2010; Trempe et al., 2012); autonomy (Wulf and Toole, 1999; Chiviacowsky and Wulf, 2002; Wu and Magill, 2011; Carter and Patterson, 2012; Fairbrother et al., 2012; Lewthwaite et al., 2015), and relatedness (Chiviacowsky et al., 2018; Gonzalez and Chiviacowsky, 2018) support for learners during practice have provided evidence of a positive impact on the acquisition of motor skills.

Feedback is visualized as information provided by an external agent related to aspects of an individual's understanding or performance (Hattie and Timperley, 2007). While the informational function of feedback in motor learning has been extensively demonstrated in the literature (for reviews, see Salmoni et al., 1984; Schmidt, 1991; Swinnen, 1996; Wulf and Shea, 2004), research on the motivational role of feedback in motor learning, especially linked with learners' need for competence, has only recently increased considerably. For instance, experiments in distinct settings have reported learners' general preference for receiving feedback mainly in order to confirm good instead of poor performance (Chiviacowsky and Wulf, 2002, 2005; Patterson and Carter, 2010; Patterson et al., 2011; Fairbrother et al., 2012; Chiviacowsky, 2014; Grand et al., 2015; Carter et al., 2016). Other studies have shown, in different tasks and populations, that deliberately affording learners with feedback after trials with relatively slight errors positively affect motivation and learning, compared with feedback provided after trials with larger errors (Chiviacowsky and Wulf, 2007; Clark and Ste-Marie, 2007; Badami et al., 2012; Saemi et al., 2012; Abbas and North, 2018). Similarly, feedback on a high degree of success in which relatively easy criteria for good performance were established for a task has been shown to enhance perceptions of competence and motor learning compared with feedback on a low degree of success, in which more difficult criteria for good performance were established (Chiviacowsky et al., 2012; Trempe et al., 2012; Chiviacowsky and Harter, 2015; Palmer et al., 2016). Even subtle wording differences in positive feedback statements, implying performance as a result of malleable rather than fixed capacities (Chiviacowsky and Drews, 2014), are able to affect motor learning. Taken together, in different lines of research, these studies demonstrate that feedback is not simply processed as “neutral” information by learners in order to minimize errors, without any affective implications. Instead, it also has an important motivational function with the ability to affect perceived competence, and motor learning.

The motivational role of feedback in motor learning has more recently been examined through another variable, temporal-comparative feedback, a type of feedback that compares outcomes for the same individual across practice trials, trial blocks, or practice sessions. Temporal comparison is considered an important source of information for competence evaluation (Miller, 1977; Brown and Middendorf, 1996; Butler, 1998; Wilson and Ross, 2000; Zell and Alicke, 2009). Acting in order to satisfy people's self-evaluation goals, temporal comparison describes the set of opinions and abilities that constitutes an individual self-description at different points in time (Albert, 1977). Higher learning and self-efficacy levels have been observed in learners receiving feedback that their performance had been enhanced with practice, compared with participants who were told that their performance had degraded over time (Chiviacowsky and Drews, 2016). This result supports previous motor learning findings on participants' competence evaluation amidst social-comparative or normative feedback; that is, by comparing the outcomes of an individual with those of others (Lewthwaite and Wulf, 2010; Wulf et al., 2010, 2012, 2014; Ávila et al., 2012; Gonçalves et al., 2018). Interestingly, Wilson and Ross (2000) observed that, when jointly provided, participants use at least as many temporal comparisons as social comparisons regarding personal attributes, with both independently influencing individuals' evaluations of their own skills.

While temporal-comparative feedback has been demonstrated to be a variable capable of affecting self-efficacy and motor learning, the lack of a control group in the Chiviacowsky and Drews (2016) study makes it impossible to know whether the positive condition enhanced motor learning or the negative condition decreased it. Thus, it remains unclear whether positive temporal-comparative feedback has the potential to enhance learning relative to a control group without any form of temporal comparison. Furthermore, no studies to date have observed the effects of temporal-comparative feedback on the learning of sport skills. Therefore, the purpose of the present study was to verify whether positive temporal-comparative feedback, informing participants that their performance is improving over time, would benefit motor learning. Since temporal comparison is considered an important source of information for competence evaluation (Brown and Middendorf, 1996; Butler, 1998; Wilson and Ross, 2000; Zell and Alicke, 2009), we deemed it important to carry such research.

In the present study, two groups of young adults were asked to practice a golf-putting task. The positive temporal-comparative feedback group received feedback suggesting that their average performance in a given block of trials was better than their average performance in the previous block, while the control group did not receive any temporal-comparative feedback. A retention test was performed 1 day later, without feedback, in order to examine motor learning effects as a function of temporal comparison. We also used a customized questionnaire to assess potential influences on participant level of enjoyment, perceived competence and pressure/tension, as a function of practice conditions. We expected that participants who received a general positive temporal-comparative feedback informing them of improvements across blocks of practice would show enhanced learning of the task than participants in the control group, who were not receiving temporal comparative feedback. As positive temporal comparison may presumably increase motivation by enhancing perceived competence (Deci and Ryan, 2000; Deci and Moller, 2005), we also expected that, after practice, participants would feel more satisfied with their performance and perhaps report greater enjoyment and a reduced level of pressure/tension relative to the control group.

## Methods

### Participants

Twenty-eight university students (14 males, 14 females) with a mean age of 23.2 years (*SD*: 6.71) participated in the experiment. The participants reported no prior experience with the experimental task, were not aware of the purpose of the study, and gave their informed consent to participate. The study was ethically approved by the university's institutional review board.

### Apparatus and Task

Participants were positioned on a level artificial-turf green (500 × 200 cm), indoors, and were asked to putt (white standard) golf balls to a horizontal target (a 2 × 2 cm square). They putted from a distance of 150 cm, and were asked to try to make the ball stop as near as possible to the target. The distance between the edge of the ball and the center of the target was used to measure putting accuracy.

### Procedure

After completing the consent form, all participants were assigned, randomly, to one of two groups (7 males and 7 females in each group), the positive temporal-comparative (PTC) feedback group and the control group, and introduced to the task. They were asked to putt the ball, making it stop as close as possible to the target. Participants of the PTC group were additionally informed that they would receive verbal general feedback on their average performance relative to their previous block of trials, at the end of the second, third, fourth, and fifth blocks of trials. All participants then performed five blocks of 10 practice trials. They did not perform familiarization or warm-up trials before the different experimental phases. After each trial, they received augmented feedback (deviation in cm) in addition to their intrinsic visual feedback. Participants of the PTC group received also false feedback suggesting that their performance was around 10, 15, 15, and 20% better (respectively, after the second, third, fourth, and fifth block of trials) than their performance in the previous block. This manipulation was based on the procedure described in a previous study (Chiviacowsky and Drews, 2016). In order to evaluate learning, all participants performed a retention test from the same distance 1 day later, consisting of 10 trials without any kind of augmented feedback. Similar to Wulf et al. (2012), at the end of practice the participants completed a customized questionnaire (see Table 1), which included concepts of the Intrinsic Motivation Inventory (McAuley et al., 1987).

**Table 1:** Results of the questionnaire completed at the end of practice (means and standard deviations). Responses for each question ranged from 0 (“not at all”) to 10 (“very”). Significant group differences are indicated by*.

**Questions**	**Temporal-comparison**	**Control**
**ENJOYMENT**		
How much did you enjoy practice this task today?	8.36 (1.50)	8.85 (1.50)
**PERCEIVED COMPETENCE**		
How satisfied are you with your performance on the golf task today?	7.57 (1.84)	5.92 (1.22) *
**PRESSURE/TENSION**		
How nervous were you while putting golf balls?	2.78 (2.81)	4.07 (3.10)

### Data Analysis

Deviations from the target were averaged across blocks of 10 trials in order to assess putting performance for the practice phase and the retention test. A 2 (groups) × 5 (blocks) analysis of variance (ANOVA) with repeated measures on the last factor was used to analyse the practice data. Separate one-way ANOVAs were used for the analysis of retention test data and questionnaire responses. Partial eta-squared values were used to indicate effect sizes for significant results (η_*p*_2) and the alpha was set at 0.05 for all analysis.

## Results

### Putting Accuracy

#### Practice

**Figure 1:**
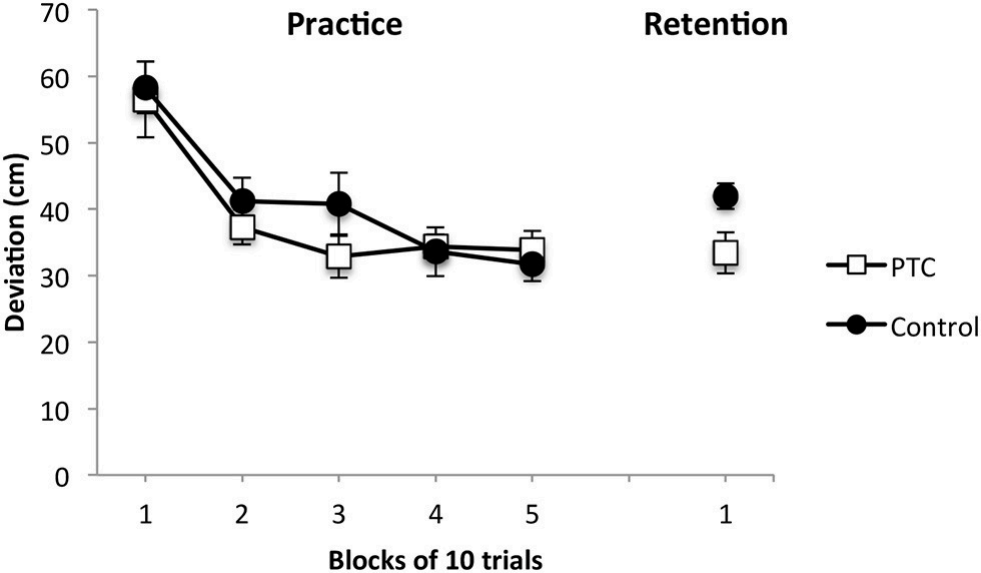
Putting performance (i.e., deviation from the center of the target) of the positive temporal-comparison and control groups during practice and retention. Error bars indicate standard errors.

During the practice phase (see Figure 1), participants in both groups reduced their deviations from the target. Block (b) means for the PTC group were: b1 = 56.5, b2 = 37.3, b3 = 32.8, b4 = 34.4, and b5 = 33.8, while block means for the control group were: b1 = 58.3, b2 = 41.2, b3 = 40.8, b4 = 36.6, and b5 = 31.6. The main effect of block was significant, *F*_(4, 104)_ = 21.22, *p* < 0.001, η_*p*_2 = 0.45. The main effect of group, *F*_(1, 26)_ = 0.43, *p* = 0.51, and the group × block interaction, *F*_(4, 104)_ = 0.85, *p* = 0.49, were not significant.

### Retention

On the retention test, deviations from the target were smaller for the PTC group (*M* = 33.42, *SD* = 11.51) compared with the control group (*M* = 41.94, *SD* = 7.10), *F*_(1, 26)_ = 5.55, *p* = *0.0*2, η_*p*_2 = 0.18 (Figure 1).

### Questionnaire Results

Following the practice phase, the groups differed in terms of how satisfied they were with their performance. Participants receiving temporal comparison feedback rated their satisfaction significantly higher than control participants, *F*_(1, 26)_ = 7.40, *p* = 0.01, η_*p*_2 = 0.22. Both groups seemed to enjoy practicing the task to a similar extent, and the group difference in this aspect was not significant, *F*_(1, 26)_ = 1.17, *p* = 0.29. Even though the temporal comparison group appeared to report a lower level of pressure/tension at the end of practice than the control group, differences were not significant, *F*_(1, 26)_ = 1.36, *p* = 0.25 (Table 1).

## Discussion

The present study was designed to examine whether temporal-comparative feedback, suggesting that participants' performance improved over time, would enhance the learning of motor skills. A previous motor learning study (Chiviacowsky and Drews, 2016) observed that participants receiving positive temporal-comparative feedback across blocks of practice demonstrated enhanced learning of a timing task compared with participants in a negative temporal-comparative feedback condition. However, it remained unknown whether this specific kind of positive feedback could benefit motor learning compared with a control condition where no temporal-comparison information was provided. Our results confirmed the hypothesis. Participants provided with positive temporal-comparative feedback showed greater learning of the golf skill, observed in the retention test, than participants in the control group. The findings are therefore in agreement with motor learning experiments manipulating competence evaluation of participants through social (e.g., Lewthwaite and Wulf, 2010; Wulf et al., 2010, 2012, 2014; Ávila et al., 2012; Gonçalves et al., 2018) or temporal-comparative feedback (Chiviacowsky and Drews, 2016).

Furthermore, while no differences were found regarding participants' enjoyment or pressure/tension levels after practice, participants in the positive temporal-comparative feedback group reported higher levels of satisfaction about their own performance compared with participants in the control group. Thus, the suggestion of a slightly higher level of standing in a task, relative to past performance, affected perceived competence among participants. The lack of difference in the participants' tension and enjoyment levels is intriguing, since these are also considered to be indicators of intrinsic motivation. However, it is not unusual to observe that different motivational categories are affected differently (e.g., Ryan, 1982; Carroll and Loumidis, 2001). A viable explanation for the benefits of receiving positive temporal-comparative feedback for motor learning, therefore, is that it creates a higher success experience for learners during practice compared with not receiving it. This success experience might be motivational for learners, improving their learning process in turn. The findings are, in this way, supportive of previous studies showing the importance of protecting learners' perceptions of competence during the motor learning process (Chiviacowsky et al., 2012; Trempe et al., 2012; Chiviacowsky, 2014; Chiviacowsky and Harter, 2015; Palmer et al., 2016).

Feeling more confident about their performance after receiving positive comparative feedback, participants in the temporal comparison group may have also created higher goals, as indicated by goal-setting and social-cognitive theories (Bandura, 1997; Bandura and Locke, 2003; Locke and Latham, 2006). Feedback has indeed been demonstrated to directly impact regulations of goal setting (Williams et al., 2000; Ilies and Judge, 2005). In the experiments of Ilies and Judge (2005), for example, participants were observed to adjust their previous goals downward or upward following negative or positive feedback, respectively, about their own performance or performance comparison with others. Their results also demonstrated that affect mediated the relationship between feedback and future goals, advancing understanding of the psychological mechanisms that learners use in interpreting and responding to feedback.

More recently, it has been proposed that conditions that provide autonomy support, enhance expectancies for performance, and induce an external focus of attention contribute to motor learning by strengthening the coupling of goals to actions, reading the motor system for task execution, helping to consolidate memories (Wulf and Lewthwaite, 2016). While informing improvements across practice blocks, temporal-comparative feedback can increase positive expectations for future performance in similar contexts, thus facilitating learning. In fact, confidence (or self-efficacy) has been revealed to predict both motor performance (Moritz et al., 2000) and motor learning (e.g., Chiviacowsky et al., 2012; Stevens et al., 2012; Chiviacowsky and Harter, 2015).

In conclusion, the findings provide the first evidence that positive temporal-comparative feedback enhances the learning of motor skills. Specifically, we demonstrate that the provision of temporal-comparative feedback can increase learners' perceptions of competence and facilitate the acquisition of golf putting. More broadly, the results highlight the motivational function of feedback in motor learning. With the potential to enhance perceived competence, positive temporal-comparative feedback may act by satisfying the individual's basic psychological needs (Deci and Ryan, 2000), increasing motivation and promoting higher motor learning. Future studies could further reveal the specific underlying mechanisms of temporal-comparison feedback, as well as its effects on the learning of different types of tasks in distinct populations. The use of additional retention (ex. 1 week) and transfer tests might also be interesting to test the persistence and adaptability of the effects. In addition, the present experiment used simple deviations from the target for data analysis. The use of other measures and methods for describing data from two-dimensional performances could provide a more comprehensive analysis of the scores (Hancock et al., 1995; Land et al., 2014). Also, while participants in the PTC group did not explicitly report awareness of the false comparative feedback used in the present study, subsequent studies could test accurate instead of bogus patterns of improvements for comparative information. Such research could provide further evidence-based answers, substantiating recommendations for practical applications. Since the comparison of individuals with their own past performance during practice usually results in progress over time, positive temporal comparative feedback may be considered an easy and useful tool for motor learning enhancement.

## Ethics Statement

This study was carried out in accordance with the recommendations of Ethical committee of Federal University of Pelotas with written informed consent from all subjects. All subjects gave written informed consent in accordance with the Declaration of Helsinki. The protocol was approved by the Ethical committee of Federal University of Pelotas.

## Author Contributions

SC, NH, GG, and PC conceived the study. NH and GG collected the data. SC analyzed the data, wrote the manuscript. All authors were responsible for the final approval of the manuscript.

### Conflict of Interest Statement

The authors declare that the research was conducted in the absence of any commercial or financial relationships that could be construed as a potential conflict of interest.
